# The price of privilege: helping mom die at home

**DOI:** 10.1093/geroni/igaa057.3183

**Published:** 2020-12-16

**Authors:** Renee Beard

**Affiliations:** College of the Holy Cross, Worcester, Massachusetts, United States

## Abstract

Americans overwhelmingly wish to age in place and many explicitly want to die at home. Yet, the anemic welfare state means that only the most fortunate among us are able to achieve that goal. A disproportionate burden of care falls squarely to families, which are smaller and more geographically spread out than ever before. Carers too often wind up in environments that are far from conducive, namely being older and perhaps frail themselves or younger and perhaps with small children of their own. Drawing on an autoethnographic study of my mother’s final years and a case study analysis of one innovative home care agency, this project examines the individual and organizational factors that allow one family to grant their family member’s wish to die at home. Grounded theory methods revealed facilitators including presence of a home-based long term care insurance policy, geographic mobility, and access to a democratically-oriented home care organization. Barriers, of course, include lack of access to long term care insurance and a daughter who lives in a progressive state with a waiver for Home and Community Based Services. While the privilege of access underscores the social determinants of aging, this case study reveals some important features that suggest how senior social services could be. Even for the “ideal type” presented here, the many trials and tribulations of aiding a loved one to die at home relate to the untenable nature of doing it all in a context whereby social services are fragmented and driven by financial incentives.

